# Cannabidiol Inhibits Multiple Ion Channels in Rabbit Ventricular Cardiomyocytes

**DOI:** 10.3389/fphar.2022.821758

**Published:** 2022-02-03

**Authors:** Dmytro Isaev, Waheed Shabbir, Ege Y. Dinc, Dietrich E Lorke, Georg Petroianu, Murat Oz

**Affiliations:** ^1^ Department of Cellular Membranology, Bogomoletz Institute of Physiology, Kiev, Ukraine; ^2^ Department of Medicine, Division of Nephrology and Cellular and Molecular Pharmacology, University of California, San Francisco, San Francisco, CA, United States; ^3^ Department of Neurology, Diskapi Training and Research Hospital, Ankara, Turkey; ^4^ Department of Anatomy and Cellular Biology, College of Medicine and Health Sciences, Khalifa University, Abu Dhabi, United Arab Emirates; ^5^ Center for Biotechnology, Khalifa University of Science and Technology, Abu Dhabi, United Arab Emirates; ^6^ Department of Pharmacology and Therapeutics, College of Medicine and Health Sciences, Khalifa University, Abu Dhabi, United Arab Emirates; ^7^ Department of Pharmacology and Therapeutics, Faculty of Pharmacy, Kuwait University, Safat, Kuwait

**Keywords:** cannabidiol, cannabinoid, ion channels, rabbit, ventricular myocytes

## Abstract

Cannabidiol (CBD), a major non-psychotropic cannabinoid found in the Cannabis plant, has been shown to exert anti-nociceptive, anti-psychotic, and anti-convulsant effects and to also influence the cardiovascular system. In this study, the effects of CBD on major ion currents were investigated using the patch-clamp technique in rabbit ventricular myocytes. CBD inhibited voltage-gated Na^+^ and Ca^2+^ channels with IC_50_ values of 5.4 and 4.8 µM, respectively. In addition, CBD, at lower concentrations, suppressed ion currents mediated by rapidly and slowly activated delayed rectifier K^+^ channels with IC_50_ of 2.4 and 2.1 µM, respectively. CBD, up to 10 μM, did not have any significant effect on inward rectifier *I*_K1_ and transient outward *I*_to_ currents. The effects of CBD on these currents developed gradually, reaching steady-state levels within 5–8 min, and recoveries were usually slow and partial. Hill coefficients higher than unity in concentration-inhibition curves suggested multiple CBD binding sites on these channels. These findings indicate that CBD affects cardiac electrophysiology by acting on a diverse range of ion channels and suggest that caution should be exercised when CBD is administered to carriers of cardiac channelopathies or to individuals using drugs known to affect the rhythm or the contractility of the heart.

## Introduction

Cannabis cultivars have been used since ancient times for recreational and medicinal purposes. Cannabis plant extracts contain over 100 structurally related, highly lipophilic terpenoid derivatives, collectively designated as phytocannabinoids (Pertwee et al., 2010). Among these phytocannabinoids, cannabidiol (CBD) is of great pharmacological interest because of its lack of potency on cannabinoid type 1 and type 2 receptors that are thought to mediate the psychotropic activity of the cannabis plant, an effect mainly mediated by (-)-*trans*-Δ^9^-Tetrahydrocannabinol (Mechoulam and Hanus, 2002; Pertwee et al., 2010). Cannabidiol has been shown to exert anti-nociceptive, anti-psychotic, and anti-convulsant effects (Hill et al., 2012; Vitale et al., 2021). More recently it has been approved by the US Food and Drug Administration for the treatment of patients with refractory epilepsy, e.g., Lennox–Gastaut and Dravet syndromes (Vitale et al., 2021). However, the specific molecular and cellular mechanisms underlying the effects of CBD remain largely unknown.

In earlier studies, CBD, in the concentration range of 1–30 μM, has been shown to modulate the functions of ligand-gated ion channels including nicotinic α_7_ (Mahgoub et al., 2013), 5-HT_3_ (Yang et al., 2010), glycine (Ahrens et al., 2009), and GABA_A_ (Bakas et al., 2017) receptors as well as voltage-gated Na^+^ (Ghovanloo et al., 2018), L-type Ca^2+^ (Ali et al., 2015), T-type Ca^2+^ (Ross et al., 2008) and K^+^ (Orvos et al., 2020) channels. Drugs interacting with such a large repertoire of ion channels can legitimately be suspected to cause cardiac safety concerns due to their arrhythmogenic potential. Recently, CBD was found to affect action potential durations (Ali et al., 2015; Le Marois et al., 2020; Orvos et al., 2020; Topal et al., 2021) by acting on cardiac Na^+^ (Le Marois et al., 2020; Orvos et al., 2020), L-type Ca^2+^ (Ali et al., 2015; Le Marois et al., 2020) and K^+^ (Le Marois et al., 2020; Orvos et al., 2020; Topal et al., 2021) channels in rat, rabbit, and dog cardiomyocytes and in heterologous expression systems, such as HEK293 and COS cells. In this study, we investigated the effects of CBD on voltage-gated K^+^, Na^+^ and Ca^2+^ channels in rabbit ventricular cardiomyocytes, as a single assay system, and discussed our findings comparatively.

## Methods

Male New Zealand white rabbits (ca. 2–2.5 kg) were anaesthetized by injection of sodium pentobarbitone (40 mg/kg) into a marginal ear vein and killed by removal of the heart.

This study was carried out in accordance with the recommendations in the Guide for the Care and Use of Laboratory Animals of the National Institutes of Health and approved by the Animal Care Committee of Bogomoletz Institute of Physiology of National Academy of Science of Ukraine. Ventricular myocytes were prepared by slight modification of the method described earlier (Al Kury et al., 2014a; Ali et al., 2015). Briefly, hearts were mounted for retrograde perfusion according to the Langendorff method and perfused at a constant flow of 10 ml g heart^−1^ min^−1^ and at 37°C with a solution containing (mM): 130 NaCl, 5.4 KCl, 1.4 MgCl_2_, 0.75 CaCl_2_, 0.4 NaH_2_PO_4_, 5 HEPES, 10 glucose, 20 taurine, and 10 creatine set to pH 7.3 with NaOH. When the heart had stabilized, perfusion was continued for 4 min with Ca^2+^-free isolation solution containing 0.1 mM EGTA, and then for 6 min with cell isolation solution containing 0.05 mM Ca^2+^, 0.8 mg/ml collagenase (type 1; Worthington Biochemical Corp, United States) and 0.075 mg/ml protease (type X1 V; Sigma, Taufkirchen, Germany). Ventricles were excised from the heart, minced and gently shaken in collagenase-containing isolation solution supplemented with 1% BSA. Cells were filtered from this solution at 4 min intervals and resuspended in isolation solution containing 0.75 mM Ca^2+^.

### Whole Cell Patch-Clamp Technique

Myocytes were dispersed and allowed to settle for at least 1 h at room temperature (22–24°C) prior to their use. Measurements were performed only in quiescent myocytes with clear-cut striations. The whole-cell patch-clamp technique was used to evaluate individual ionic currents using an Axopatch 200B amplifier (Molecular Devices, Downington, PA, United States) linked to an A/D interface (Digidata 1,322; Molecular Devices). Currents were filtered at 5 kHz and acquired using Axon pCLAMP 8.2 (Molecular Devices, Downington, PA, United States). Heat-polished borosilicate glass pipettes (World Precision Instruments, Sarasota, FL, United States) with tip resistance of 1–3 MΩ were used to establish GΩ seals and continuity with the intracellular medium.

Cell capacitance (C_m_) was calculated by integrating the area under an uncompensated capacity transient elicited by a 10 mV depolarizing pulse from a holding potential of −80 mV.

The total series resistance (R_s_) between the pipette interior and the cell membrane in the whole-cell configuration was calculated from the estimates of C_m_ and the time constant (τ_c_) of the capacitative current decay using the equation τ_c_ = R_s_ x C_m_. The mean R_s_ for the pathway between the pipette and the cell membrane after rupture of the membrane seal was calculated to be 2.36 ± 0.09 MΩ. After establishment of whole-cell configuration and measurement of C_m_, the R_s_ was compensated by 50–60%. Junction potentials under our conditions were approximately −3 mV and were not corrected.

The dialyzing internal pipette contained (in mM): 135 KCl, 10 NaCl, 10 HEPES, 5 Mg-ATP, 10 μM cyclic AMP; titrated with KOH to pH 7.2. The control perfusate was a modified Tyrode solution containing (in mM): 137 NaCl, 5.4 KCl, 1 MgCl_2_, 2 CaCl_2_, 10 HEPES, 10 glucose; titrated with NaOH to pH 7.4. Extracellular solution for recordings of Na^+^ currents consisted of (in mM): 100 TEACl, 40 NaCl, 10 glucose, 1 MgCl_2_, 5 CsCl, 0.1 CaCl_2_, 10 HEPES [adjusted to pH 7.3 with CsOH; (Al Kury et al., 2014b)], and 10 µM nifedipine included to suppress L-type Ca^2+^ current. Intracellular solution contained (in mM) 135 CsCl, 5 NaCl, 10, EGTA, 10 HEPES, and 1 MgATP (adjusted to pH 7.25 with CsOH). For the recording of Ca^2+^ currents, the whole-cell bath solution contained (in mM): 95 NaCl, 50 TEACl, 2 MgCl_2_, 2 CaCl_2_, 10 HEPES and 10 glucose (adjusted to pH 7.35 with NaOH). The pipette solution contained (in mM): 140 CsCl, 10 TEACl, 2.0 MgCl_2_, 2 HEPES 1 MgATP and 10 EGTA (adjusted to pH 7.25 with CsOH). For recording K^+^ currents, the external solution contained (in mM) 140 N-methyl-D-glucamine, 5.4 KCl, 0.1 CaCl_2_, 1 MgCl_2_, 0.5 CdCl_2_, 10 glucose, and 10 HEPES (pH 7.3 with NaOH). The pipette solution for *I*_K_ recordings contained (in mM) 120 potassium glutamate, 20 KCl, 2 MgCl_2_, 10 HEPES, 5 EGTA, 2 Mg-ATP, and 2 QX314; adjusted to pH 7.4 with KOH (Rose et al., 2005). When *I*_Kr_ was recorded, *I*_Ks_ was inhibited by using 1 µM selective *I*_Ks_ blocker HMR 1556 (Tocris, Minneapolis, MN, United States). During *I*_Ks_ measurements, *I*_Kr_ was blocked by 1 µM dofetilide (Sigma, St. Louis, MO, United States), and the bath solution contained 0.1 µM forskolin (Sigma, St. Louis, MO, United States). For recordings of *I*_to_, 0.5 mM CdCl_2_ and 20 µM tetrodotoxin (TTX) were included to eliminate *I*_L-Ca_ and *I*_Na_, respectively. In pharmacological verification of Na^+^ and Ca^2+^ currents, bath applications of nifedipine (10 μM; n = 4) and TTX (20 μM, n = 4) completely inhibited *I*_L-Ca_ and *I*_Na_, respectively. Experiments were performed at room temperature (22–24°C). Changes of external solutions and application of drugs were performed using a multi-line perfusion system with a common outflow connected to the recording chamber. A perfusion rate of 2 ml/min was used routinely in a recoding chamber with a volume of 200 µl.

Cannabidiol (CBD) was from Sigma (St. Louis, MO, United States). It was dissolved in 100% DMSO, and final concentrations were diluted from stock solutions. Stocks were kept at −20°C until their use. The control solution also contained the same quantities of the stock vehicle (e.g., 0.0001% of DMSO v/v at 1 μM of CBD). Reagents and chemicals used in our experiments were purchased from Sigma-Aldrich (St. Louis, MO, United States).

#### Statistical Analysis

All cumulative results are expressed as mean ± SEM as indicated.

Statistical significance among groups was determined using pair-wise comparisons (Mann-Whitney *U*-Test) followed by Bonferroni Post-hoc analysis. Statistical analysis of the data was performed using Origin 7.0 software (OriginLab Corp, Northampton, MA, United States). Currents were measured in each cell before and after exposure to the drug. The number of tested cells for each type of ion current varied between 5 and 14. *p* < 0.05 was considered statistically significant.

## Results

### The Effects of Cannabidiol on the Na^+^ Channels (*I*_Na_)

**FIGURE 1:**
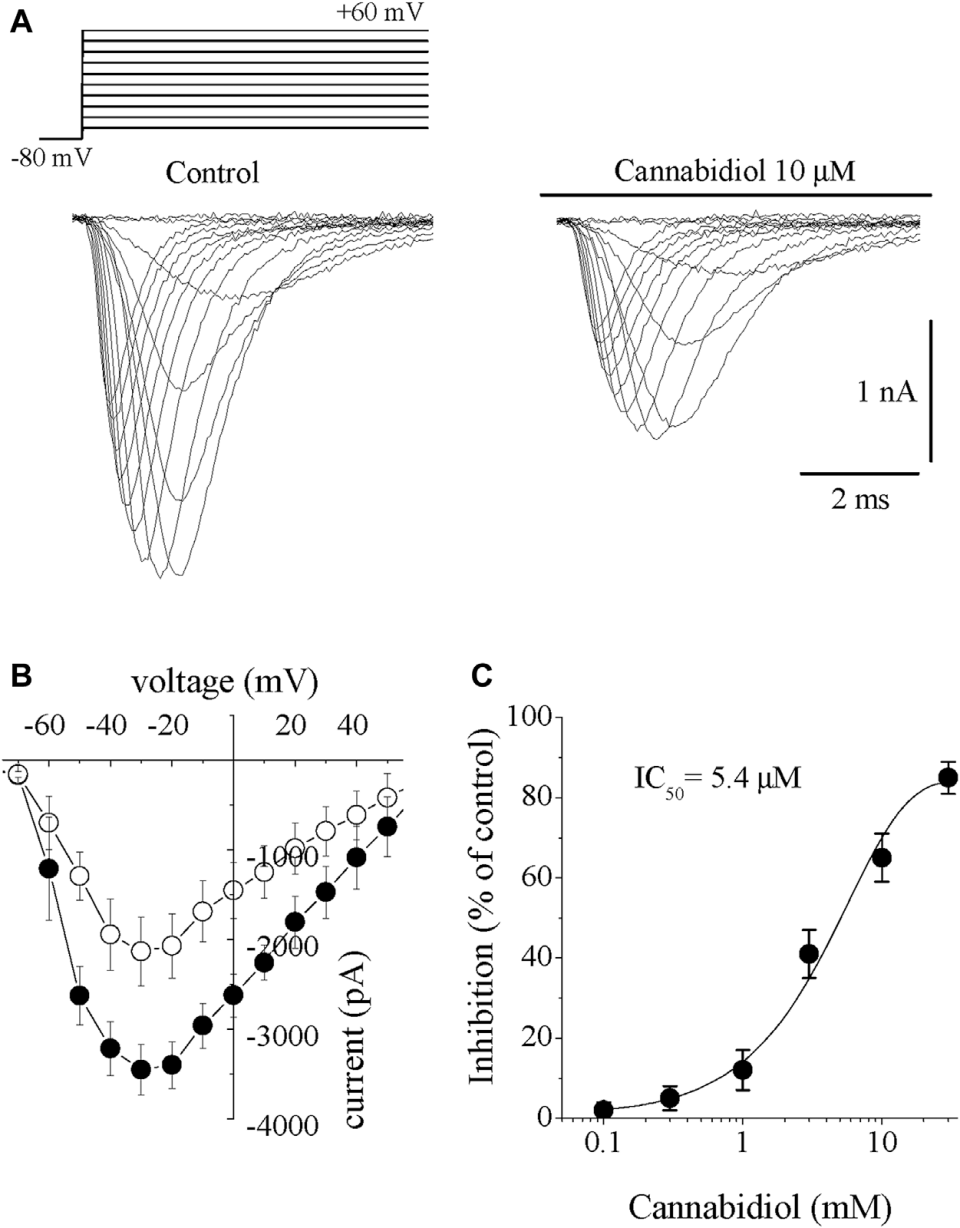
Cannabidiol suppresses the *I*_Na_ in rabbit ventricular myocytes **(A)** Original current traces elicited by voltages in the range of −60 mV to +60 mV in control conditions (on the left) and 10 min after exposure to 10 μM CBD (on the right). The pulse protocol to activate *I*_Na_ is presented in the inset **(B)** Current-voltage relations of *I*_Na_ in the absence and presence of 10 μM CBD are presented with filled and open circles, respectively (n = 6) **(C)** Concentration-inhibition curve for CBD suppression of *I*_Na_ (n = 6–8).

Inward *I*_Na_ was elicited by incremental 10 mV step depolarizations applied from a holding potential of −80 mV with 40 mМ Na^+^ outside and Cs^+^ as the major intracellular cation. *I*_Na_ started to activate at -50 mV and reached maximal amplitude at −30 mV. At more positive potentials, *I*_Na_ gradually decreased and reversed its direction at an apparent reversal potential of around +60 mV. Bath application of CBD (0.3–30 µM) caused a gradually increasing suppression of *I*_Na_. The effect of CBD was detectable at 2–3 min and reached a steady-state level within 10–15 min (n = 6). The recovery was partial during the experiments lasting up to 20–25 min. Traces of *I*_Na_ within the voltage range of −80 mV to +60 mV are presented before (control) and 10 min after 10 µM CBD in Figure 1A. Cannabidiol inhibited *I*_Na_ without causing significant changes in the *I*-*V* relationship. The current–voltage (*I*-*V*) relationship for *I*_Na_ is illustrated in Figure 1B. Cannabidiol inhibited *I*_Na_ without changing the threshold, peak and reversal potentials. The concentration-response curve is presented in Figure 1C. The IC_50_ value and Hill coefficient were 5.4 µM and 2.6, respectively (n = 6–8).

### The Effects of Cannabidiol on the L-type Ca^2+^ Channels (*I*_L-Ca_)

**FIGURE 2:**
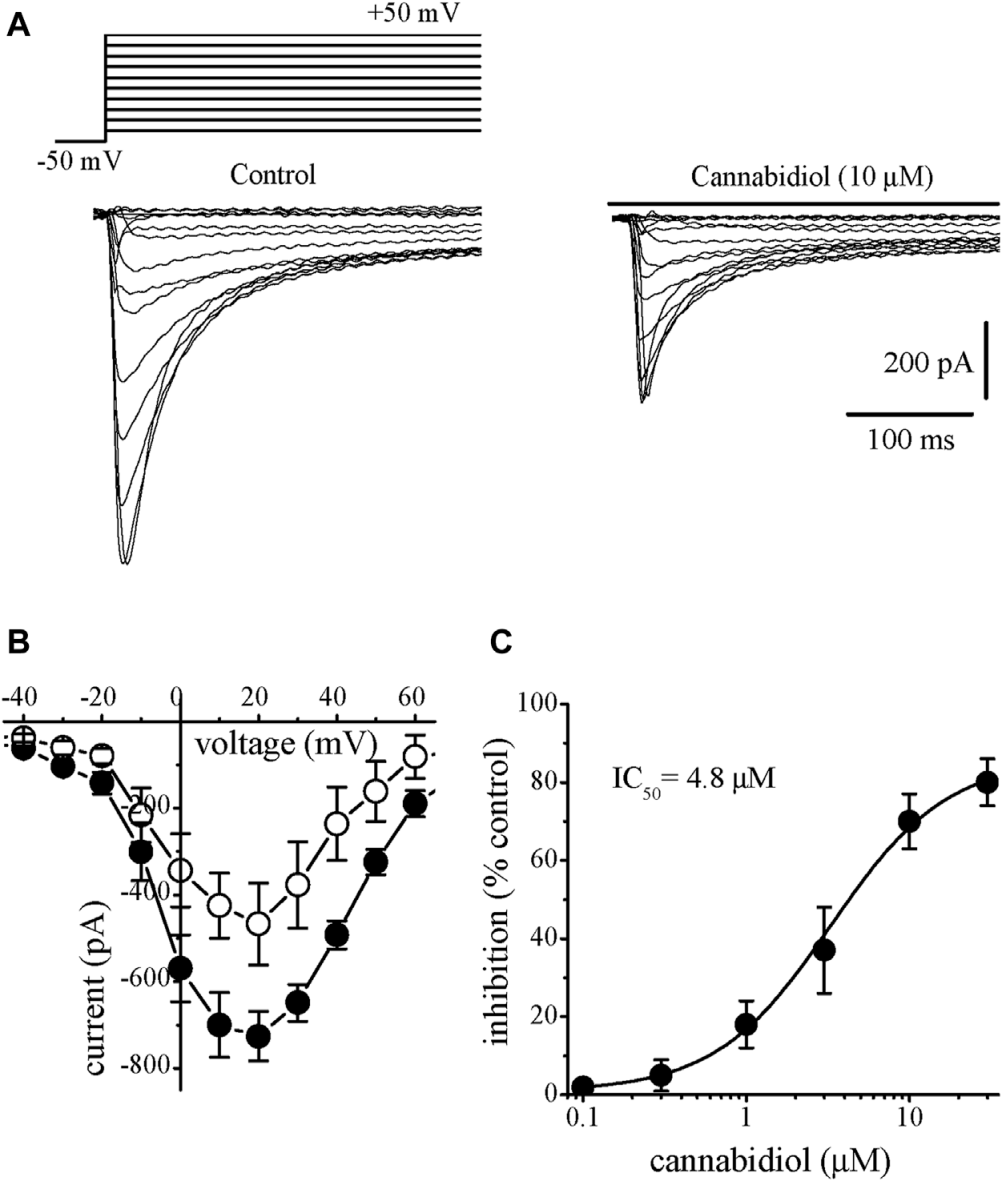
Cannabidiol inhibits the *I*_L-Ca_ in rabbit ventricular myocytes **(A)** Original current traces elicited by voltages in the range of −50 mV to +50 mV in control conditions (on the left) and after 10 min exposure to 10 μM CBD (on the right). The pulse protocol to evoke *I*_L-Ca_ is presented in the inset **(B)** Current-voltage relations of *I*_L-Ca_ in the absence and presence of 10 μM CBD are presented with filled and open circles, respectively (n = 6) **(C)** Concentration-inhibition curve for CBD suppression of *I*_L-Ca_ (n = 5–8).

We have also investigated the effect of CBD on L-type Ca^2+^ currents (*I*_L,Ca_). *I*_L,Ca_ was recorded in the presence of intracellular Cs^+^ and extracellular TEA^+^ to suppress K^+^ currents while retaining 95 mM Na^+^ in the extracellular solution. Elimination of contaminating Na^+^ current during the recording of *I*_L,Ca_ was achieved by applying voltage step-pulses from a relatively depolarized potential of −50 mV, which produced steady-state *I*_Na_ inactivation (Al Kury et al., 2014b). Application of CBD (0.3–30 µM) caused a steadily progressing inhibition of *I*_Na_. The effect of CBD was detectable at 2–3 min and reached a steady-state level within 5–10 min (n = 6). The recovery was partial during the experiments lasting up to 20 min. Traces of current elicited in a voltage range from −40 to +60 are presented in control (on the left) and 10 min after 10 µM CBD application (Figure 2A). In response to step depolarizations, *I*_L,Ca_ had much slower kinetics and activated at more positive potentials than *I*_Na_. Inward *I*_Na_ started to appear at −30 mV, reached a maximum at around +10 mV, and approached zero at about +50 mV (Figure 2B). Cannabidiol inhibited *I*_Na_ in a concentration-dependent manner with IC_50_ value of 4.8 µM and Hill coefficient of 1.8 (n = 5–8; Figure 2C).

### The Effects of Cannabidiol on the Delayed Rectifier K^+^ Channels (*I*_K_)

In most cases the delayed rectifier K^+^ current in mammalian ventricular myocytes is composed of two components, a slowly activating *I*_Ks_, and a rapidly activating but inwardly rectifying *I*_Kr_ (Sanguinetti and Jurkiewicz, 1990).

#### Slowly Activating Delayed Rectifier, *I*_Ks_

**FIGURE 3:**
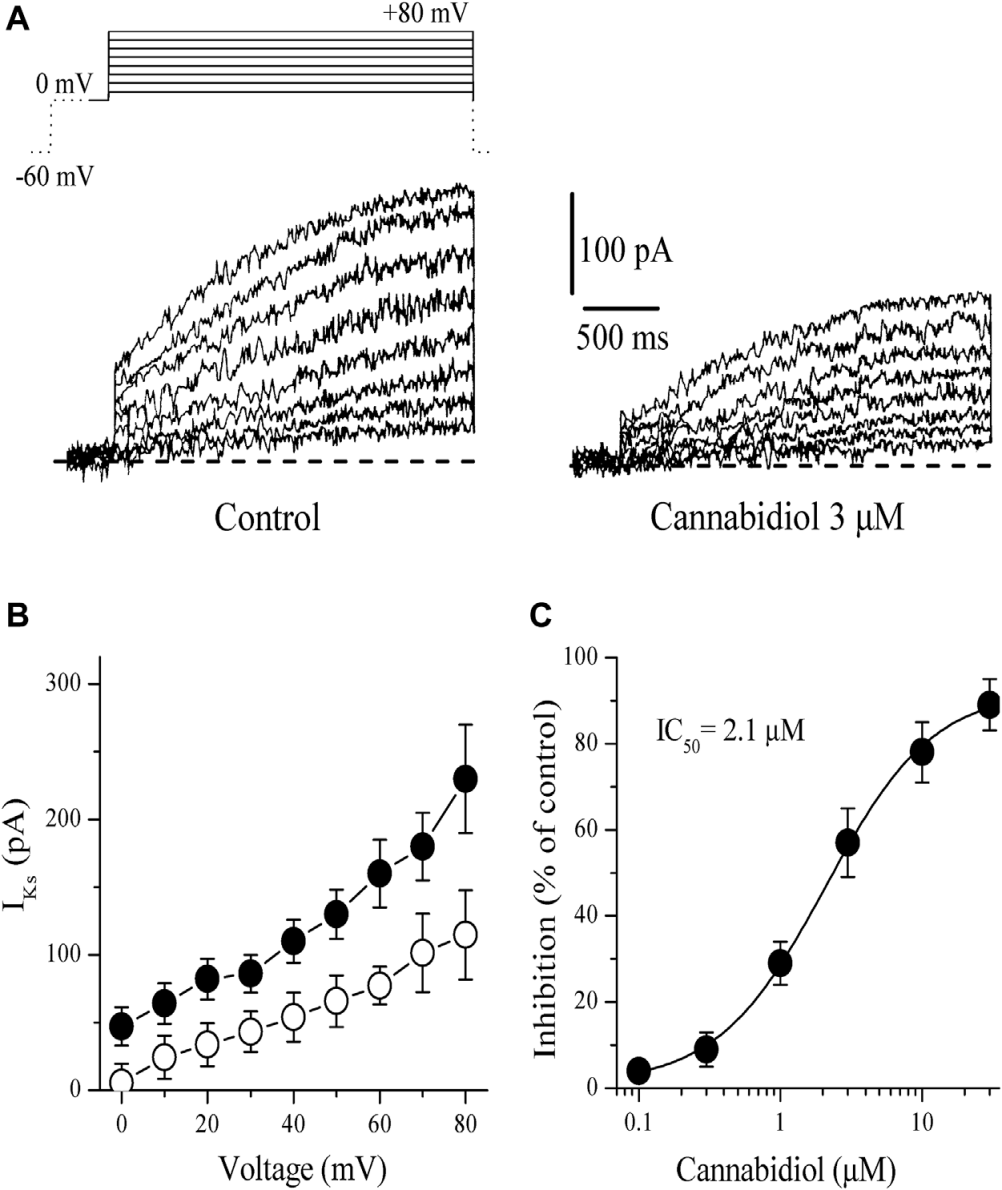
Cannabidiol suppresses the *I*_Ks_ component of the delayed rectifying *I*_K_ **(A)** Original current traces recorded in control conditions (on the left) and after 10 min exposure to 3 μM CBD (on the right) in rabbit cardiomyocytes. The pulse protocol to activate *I*_Ks_ is presented in the inset. Only solid lines in the voltage protocols are presented in current traces. Dotted line indicates 0 mV baseline **(B)** Current-voltage relations of *I*_Ks_ in the absence and presence of 3 μM CBD are presented with filled and open circles, respectively (n = 5) **(C)** Concentration-inhibition curve for CBD inhibition of *I*_Ks_ (n = 4–7).

The slowly activating delayed-rectifier K^+^ current (*I*_Ks_) was measured as the time-dependent current accompanying 2.5 s pulses from 0 to +80 mV following a 1 s conditioning pulse from −60 to 0 mV to activate and inactivate *I*_Na_ and *I*_Ca_. Application of CBD (0.1–10 µM) caused a gradually developing inhibition of *I*_Ks_. The effect of CBD was detectable at 2–3 min and reached a steady-state level within 5–10 min. Figure 3A presents current traces recorded between 0 mV and +80 mV in control (on the left) and 10 min after 3 µM CBD application. Figure 2B presents the effect of 10 µM CBD on the current-voltage relationship of *I*_Ks_ (n = 6). The effect of CBD on *I*_Ks_ was not voltage-dependent as it suppressed *I*_Ks_ to the same extent at all potentials. Concentration-dependent inhibition of *I*_K_ is presented in Figure 3C. The IC_50_ value and Hill coefficient were 2.1 µM and 1.7, respectively (n = 4–7).

#### Rapidly Activating Delayed Rectifier, *I*_Kr_

**FIGURE 4:**
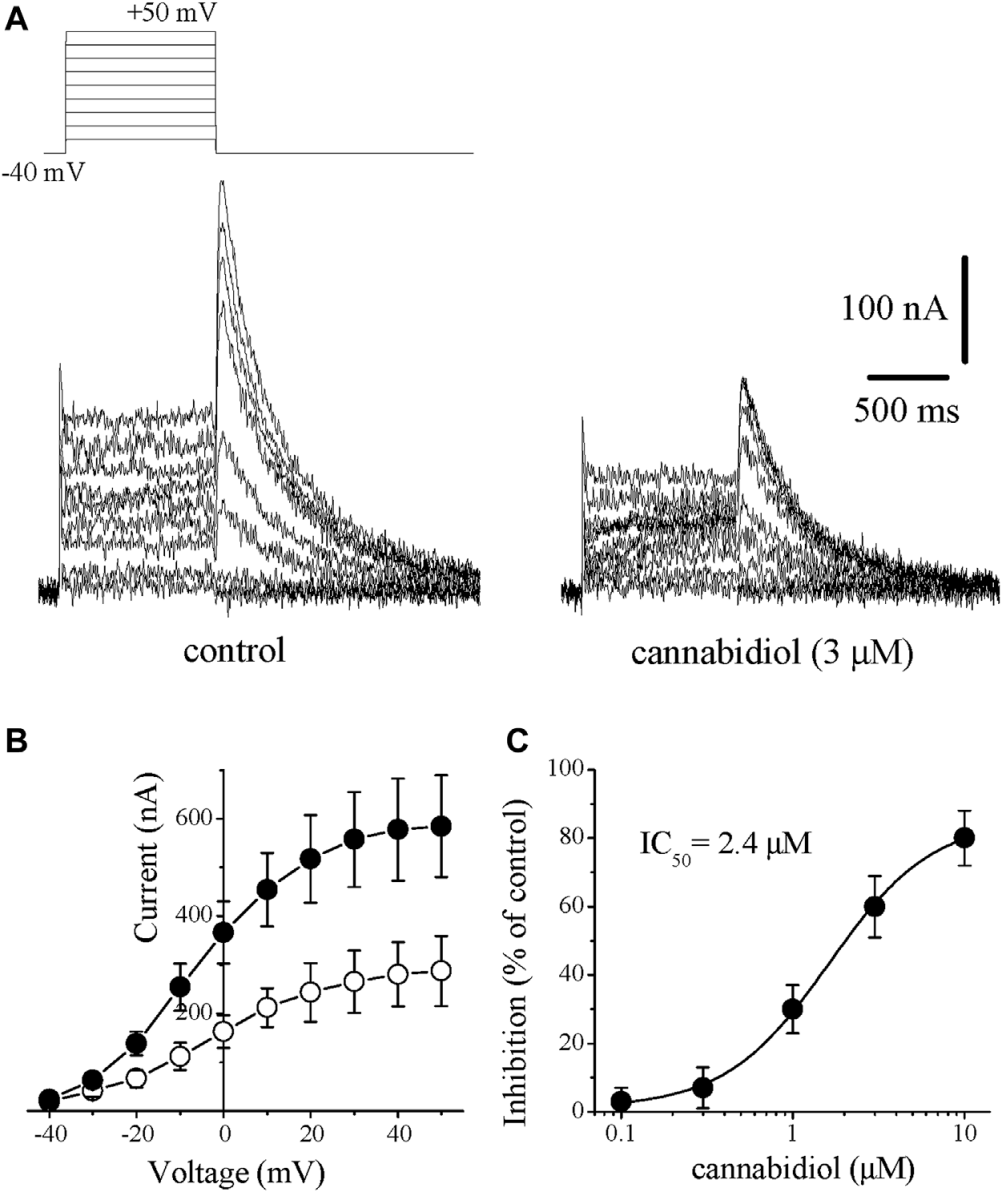
Cannabidiol suppresses the *I*_Kr_ tail current. Original traces of currents recorded in rabbit cardiomyocytes are presented in panel **(A)**. *I*_Kr_ tails were measured as a time-dependent component of the tail current activated in response to membrane repolarization. Recordings in control and after 10 min in the presence of 3 μM CBD are presented on the left and right panels, respectively. The pulse protocol to evoke *I*_Kr_ is presented in the inset **(B)** Current-voltage relation showing the cumulative effect of 3 μM CBD. Data points represent the averages of currents recorded with the same protocol described above in 6 cells before (control, filled circles) and after (open circles) CBD application **(C)** Concentration-inhibition curve for CBD inhibition of *I*_Kr_ tails (n = 5–8).

The other member of the delayed rectifier family, *I*_Kr_ was quantified by activating *I*_Kr_ in the range of −40 mV and +50 mV and measuring the deactivating tail currents in response to repolarizing test pulse of −40 mV in rabbit cardiomyocytes. *I*_Kr_ tails were measured as the time-dependent currents which reached to maximal at +30 mV upon repolarizing the membrane −40 mV from positive potentials. The difference in currents measured (peak of the tail current minus the current at the beginning of test potential) was taken as an estimate of *I*_Kr_. In order to increase the proportion of *I*_Kr_, we inhibited *I*_Ks_ by 1 µM of selective *I*_Ks_ blocker HMR 1556 and omitted cAMP and Ca^2+^ from the solution to further suppress the contribution of the *I*_Ks_ component. Bath application of CBD (0.1–10 µM) caused inhibition of *I*_Kr_ which was detectable at 2–3 min and reached a steady-state level within 5 min. Traces of currents elicited at voltages ranging from −40 to +50 mV are shown in the absence (control) and 10 min presence of 3 μM CBD in Figure 4A, effects of CBD on the *I-V* relationship of *I*_Kr_ are presented in Figure 4B (n = 7). The concentration-dependent effect of CBD is shown in Figure 4C. The IC_50_ value and Hill coefficient were 2.4 µM and 2.7, respectively (n = 5–8).

### The Effect of Cannabidiol on Transient Outward K^+^ Current (*I*_to_)

**FIGURE 5:**
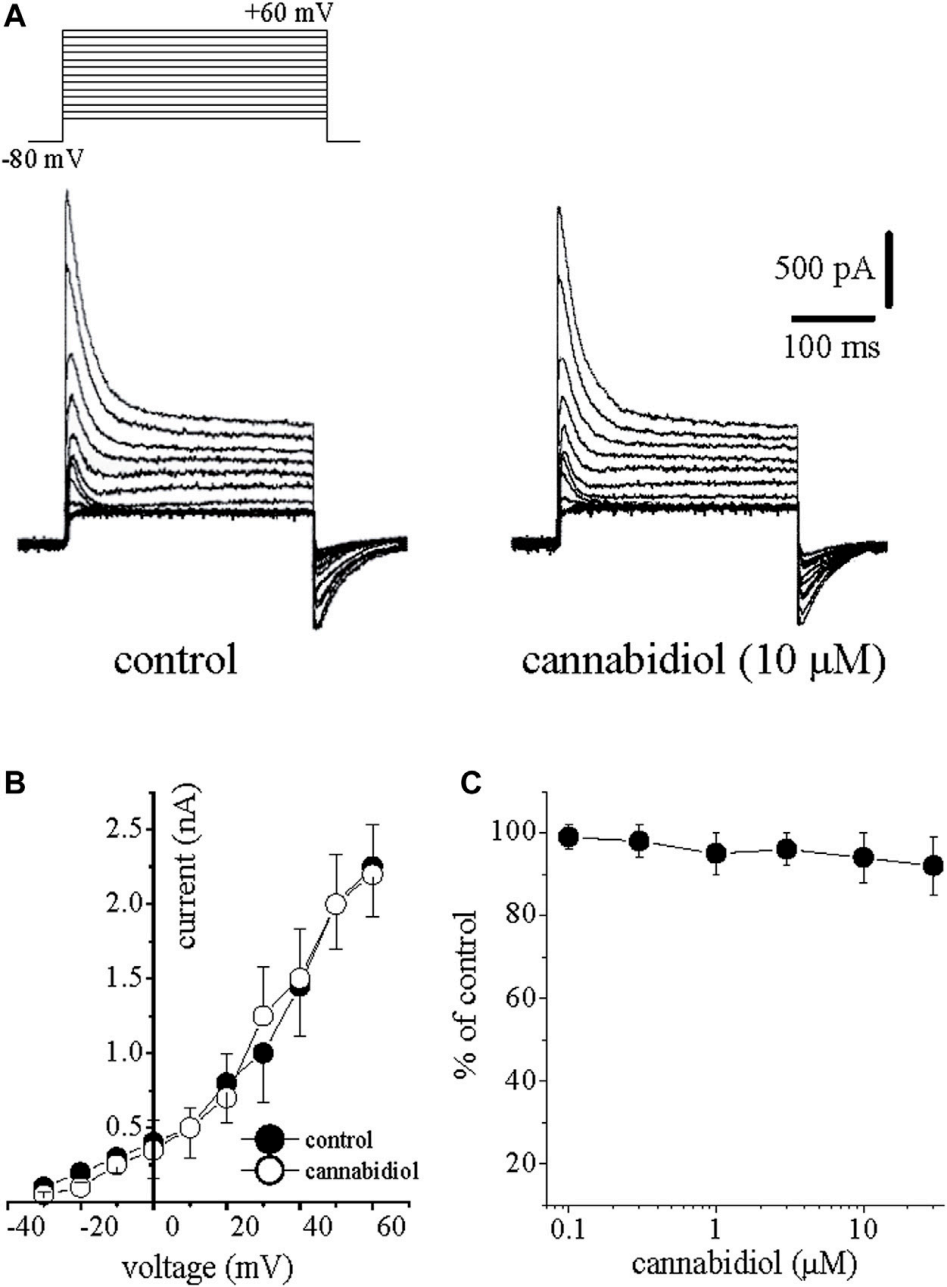
The effects of cannabidiol on the transient outward *I*_to_ in rabbit ventricular myocytes **(A)** Original traces of *I*_to_ obtained in response to incremental depolarizations from a holding potential of −80 mV up to +60 mV are presented in controls (on the left) and after 10 min exposure to 10 μM CBD. The pulse protocol to activate *I*_to_ is presented in the inset **(B)** Averages of current-voltage relation of *I*_to_ in the absence (filled circles) and presence of CBD (open circles) are presented (n = 6) **(C)** The effect of increasing CBD concentrations on *I*_to_ (n = 4–6).

Using 300 ms test pulses from −80 mV to +60 mV applied at 5 s intervals, *I*_to_ was defined as the initial transient peak of the current minus the maintained current at the end of the pulse. Application of CBD up to 10 µM did not cause a significant effect on the amplitudes and kinetics of *I*_to_. Current traces activated in the range of −40 mV to +60 mV in control (on the left) and 10 min after 10 µM CBD applicated are shown in Figure 5A. The *I-V* relationships of *I*_to_ in the absence and presence of 10 µM CBD are presented in Figure 5B (n = 6). Figure 5C demonstrates the lack of a CBD effect on *I*_to_ (n = 4–6 cells).

### The Effect of Cannabidiol on the Inward Rectifier K^+^ Current (*I*_K1_)

**FIGURE 6:**
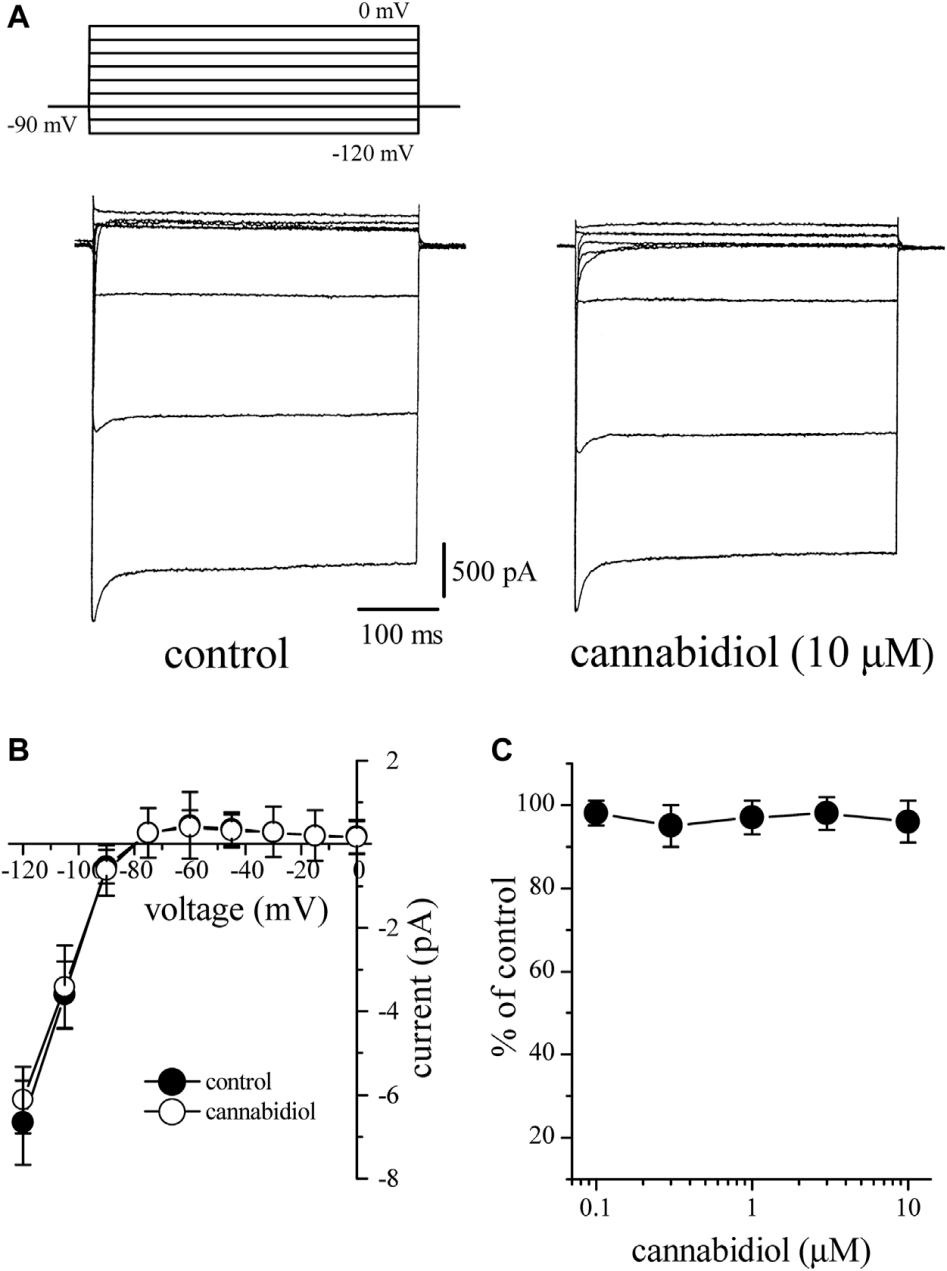
Cannabidiol does not have a significant effect on *I*_K1_ **(A)** Original current traces recorded in control conditions (on the left) and after10 min exposure to 10 μM CBD (on the right) in a rabbit cardiomyocyte. The pulse protocol to evoke *I*_K1_ is presented in the inset **(B)** Current-voltage relations of *I*_K1_ in the absence and presence of 10 μM CBD are presented with filled and open circles, respectively (n = 6) **(C)** The effect of increasing CBD concentrations on *I*_K1_ (n = 4–6).

*I*_K1_ was activated from a holding potential of −90 mV to test potentials between −120 and 0 mV. The outward component of *I*_K1_ reached a maximum at around −50 mV generating a negative slope between −50 and −10 mV (Figure 6B). The amplitude of *I*_Kl_ at each voltage was determined by measuring the peak current relative to zero current. After 10 min bath application, CBD up to 10 μM had little effect on *I*_K1_ at voltages between 0 and −120 mV. The *I-V* relationships of *I*_K1_ in the absence and presence of 10 µM CBD are presented in Figure 6B (4–5). Figure 6C shows the lack of a CBD effect on the *I*_K1_ (n = 3–5 cells).

## Discussion

The main finding of this study is that CBD appears to be particularly effective in suppressing the delayed rectifier currents *I*_Kr_ and *I*_Ks_. At higher concentrations, CBD inhibits inward Na^+^ and L-type Ca^2+^ channels. On the other hand, the transient outward current *I*_to_ and inward rectifier *I*_K1_ are significantly less sensitive to CBD.

In earlier studies, action potential duration (APD) was either increased in guinea pig, rabbit, and dog cardiomyocytes (Orvos et al., 2020; Topal et al., 2021) or decreased in rat ventricular myocytes (Ali et al., 2015) and rabbit Purkinje fibers (Le Marois et al., 2020). Although action potential measurements were not conducted in the present study, our results indicate that inhibitory effects of low CBD concentrations on outward K^+^ conductances are counteracted by the inhibition of Na^+^ and Ca^2+^ conductances at high concentrations of CBD, and indirectly confirm the finding of earlier studies showing the increased duration of action potentials at relatively low CBD concentrations and no change of the duration at higher CBD concentrations in rabbit papillary muscle cardiomyocytes (Orvos et al., 2020). In the present study, CBD inhibited outward *I*_Kr_ and *I*_Ks_ currents with *I*C_50_ values of 2.4 and 2.1 µM, respectively, and inward *I*_Na_ and *I*_L-Ca_ with 4.8 and 5.4 µM. The potency of CBD observed in our study was slightly higher than the ones reported in earlier studies (Orvos et al., 2020; Topal et al., 2021), and it is likely due to temperature differences between the present study (room temperature) and earlier work (at 37°C). For example, CBD inhibition of *I*_Na_ has been shown to be significantly increased at lower temperatures (Ghovanloo et al., 2018). Similarly, the potency of CBD on *I*_L-Ca_ [at room temperature, IC_50_ ≈ 1 μM; (Ali et al., 2015)] is significantly higher than the one recorded at 37°C [IC_50_ > 10 μM; (Orvos et al., 2020)].

**FIGURE 7:**
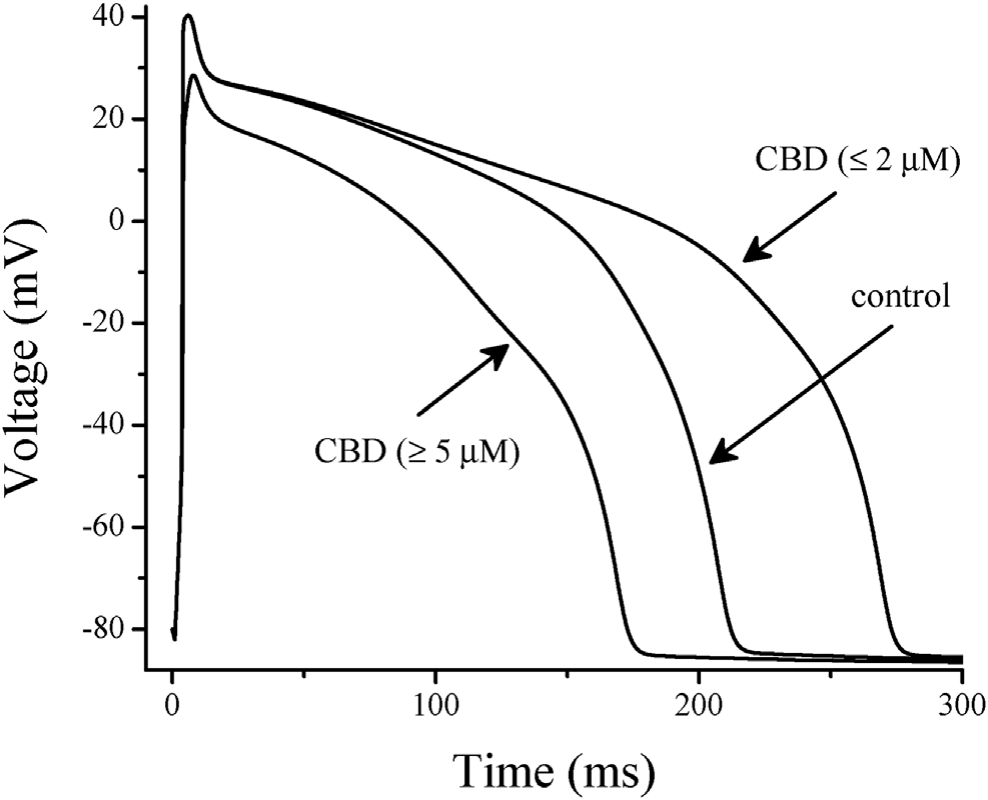
Effects of low and high concentrations of CBD on the simulated action potentials using LabHEART simulations. At low (≤2 µM) CBD concentrations, inhibition of *I*_Kr_ and *I*_Ks_ was incorporated into the model. At high (≥5 µM) CBD concentrations, inhibitions of *I*_Kr_, *I*_Ks_, *I*_Na_, and *I*_L-Ca_ were incorporated into the model. At low concentrations, CBD induced a marked prolongation (29%) of action potential duration (APD), while at high concentrations, it significantly shortened (27%) the APD.

Simulation with LabHEART, a computer model of rabbit ventricular action potentials (Puglisi and Bers, 2001), revealed that suppression of *I*_Ks_ and *I*_Kr_ alone at low (≤2 µM) concentrations of CBD as expected, causes a marked prolongation (29%) of APD, whereas integration of inhibition of *I*_Na_ and *I*_L-Ca_ at higher CBD concentrations (≥5 µM) results in 27% shortening of APD (Figure 7), which is qualitatively in agreement with experimental data in rat and rabbit cardiomyocytes (Ali et al., 2015; Le Marois et al., 2020; Orvos et al., 2020; Topal et al., 2021). Both increase and decrease of APD can potentially have arrhythmogenic effects depending mainly on the underlying pathophysiological mechanisms. Our LabHEART simulation of rabbit cardiomyocytes paced at 5 Hz indicates that both low and high concentrations of CBD may induce arrhythmic effects (Supplementary Figure S1). On the other hand, CBD has been shown to suppresses ischemia-induced ventricular arrhythmias (Walsh et al., 2010; Gonca and Darıcı, 2015) and exert cardioprotective effects in several earlier studies (for a review (Kicman and Toczek, 2020)). Importantly, the inhibition of L-type Ca^2+^ channels is likely to cause the negative inotropic effects of CBD observed in earlier studies (Ali et al., 2015; Kicman and Toczek, 2020). In line with these reports, high, but not low, concentrations of CBD markedly depressed intracellular Ca^2+^ transients, and tension development in LabHEART simulation (Supplementary Figure S2).

Plasma concentrations of CBD following intraperitoneal, oral, and intravenous administrations have been studied previously (Lodzki et al., 2003; Varvel et al., 2006; Deiana et al., 2012). Commonly used doses of CBD (3–30 mg/kg I.V.; mouse) are found to promote mean plasma CBD levels of 0.42–11.8 μM, respectively (Varvel et al., 2006). In another study, following oral and intraperitoneal CBD administration (120 mg/kg; rat), maximal plasma levels of CBD were 6.4 and 8.3 µM, respectively. Since CBD is a highly lipophilic compound with a LogP (octanol–water partition coefficient) value ranging between 6 and 8 (Lodzki et al., 2003), its membrane concentration is expected to be considerably higher than blood levels. In an earlier study, it has been shown that perfusion of isolated rat hearts with buffer containing [^3^H]-CBD results in strong accumulation of radioactivity in the tissue (Smiley et al., 1976). Therefore, the functional modulation of ion channels by the concentration ranges demonstrated in this study (2.3–5.4 µM) is likely to be pharmacologically relevant.

It is likely that CBD, a highly lipophilic agent, first dissolves into the lipid membrane and then diffuses into a non-annular lipid space to inhibit the ion channels. Consistent with this assumption, the effect of CBD on ion channels tested in this study reached a maximal level within several minutes (5–10 min) of application. Similarly, actions of several lipophilic modulators, such as capsaicin (Lundbaek et al., 2005; Alzaabi et al., 2019; Nebrisi et al., 2020), endocannabinoids (Oz et al., 2003; Spivak et al., 2007), and general anesthetics (Zhang et al., 1997; Jackson et al., 2008), on various ion channels require 5–20 min to reach their maxima, suggesting that the binding site(s) for these allosteric modifiers is/are located inside the lipid membrane and require/s a relatively slow (in minutes) time course to modulate the functions of these channels. From this aspect, it appears that alone or the combination of two mechanisms can describe the lipophilic actions of CBD (Oz, 2006; Ghovanloo and Ruben, 2021). First, CBD, like other lipophilic molecules, partitions into the lipid bilayer and alters the biophysical properties of the membrane by reducing stiffness, changing phase preference, membrane curvature and fluidity (Bach et al., 1976; Hillard et al., 1985; Ghovanloo et al., 2021). Secondly, CBD can bind directly to ion channel domains embedded in the cell membrane (Ghovanloo et al., 2021; Ghovanloo and Ruben, 2021). As a result of these mechanisms, it is likely that these hydrophobic agents, such as CBD, affect the energy requirements for gating-related conformational changes in ion channels (Spivak et al., 2007).

Collectively, our results suggest that the administration of CBD to carriers of congenital Na^+^, Ca^2+^, or K^+^ channelopathies, or its coadministration with other drugs known to affect cardiac electrophysiology should be cautioned. Moreover, the combined inhibition of Na^+^ and Ca^2+^ currents by CBD could counteract the positive inotropic effects of some heart failure drugs and could impede its use in conjunction with antiarrhythmic drugs. However, antiarrhythmic effects of CBD in ischemia-induced arrhythmia models have also been reported (Walsh et al., 2010; Gonca and Darıcı, 2015).

## Data Availability

The raw data supporting the conclusions of this article will be made available by the authors, without undue reservation.

## Supplementary Material

Supplementary Figure S1 Effects of low and high concentrations of cannabidiol on the simulated trains of action potentials elicited at stimulation frequency of 5 Hz using LabHEART simulations. **(A)** Trains of action potentials stimulated at 5 Hz under control conditions in the LabHEART simulation program. **(B)** At low (≤2 µM) CBD concentrations, inhibition of *I*_Kr_ and *I*_Ks_ was incorporated into the model. **(C)** At high (≥4 µM) CBD concentrations, inhibitions of *I*_Kr_, *I*_Ks_, *I*_Na_, and *I*_L-Ca_ were incorporated into the model. At low concentrations, CBD induced trains of incomplete partial depolarization waves, while at high concentrations, an early afterdepolarization-like arrhythmic pattern was notable.

Supplementary Figure S2 Effects of low and high concentrations of cannabidiol on the simulated intracellular Ca^2+^ transients and force development during a single twitch response in LabHEART. **(A)** At low (≤2 µM) CBD concentrations, incorporation of *I*_Kr_ and *I*_Ks_ inhibition caused a slight delay in the decay phase of Ca^2+^ transient. At high (≥4 µM) CBD concentrations, incorporations of *I*_Kr_, *I*_Ks_, *I*_Na_, and *I*_L-Ca_ inhibitions induced a marked suppression of Ca^2+^ transient. **(B)** At low (≤2 µM) CBD concentrations, incorporation of *I*_Kr_ and *I*_Ks_ inhibition caused a slight delay in the decay phase of force development. At high (≥4 µM) CBD concentrations, incorporations of *I*_Kr_, *I*_Ks_, *I*_Na_, and *I*_L-Ca_ inhibitions induced a marked inhibition of force development during a simulated twitch response of rabbit ventricular cardiomyocyte.
