# Supplementary figures and images for: Cannabidiol Inhibits Multiple Ion Channels in Rabbit Ventricular Cardiomyocytes
Source: Front Pharmacol. 2022 Feb 3;13:821758. doi: 10.3389/fphar.2022.821758 (PMC8850628; doi:10.3389/fphar.2022.821758)

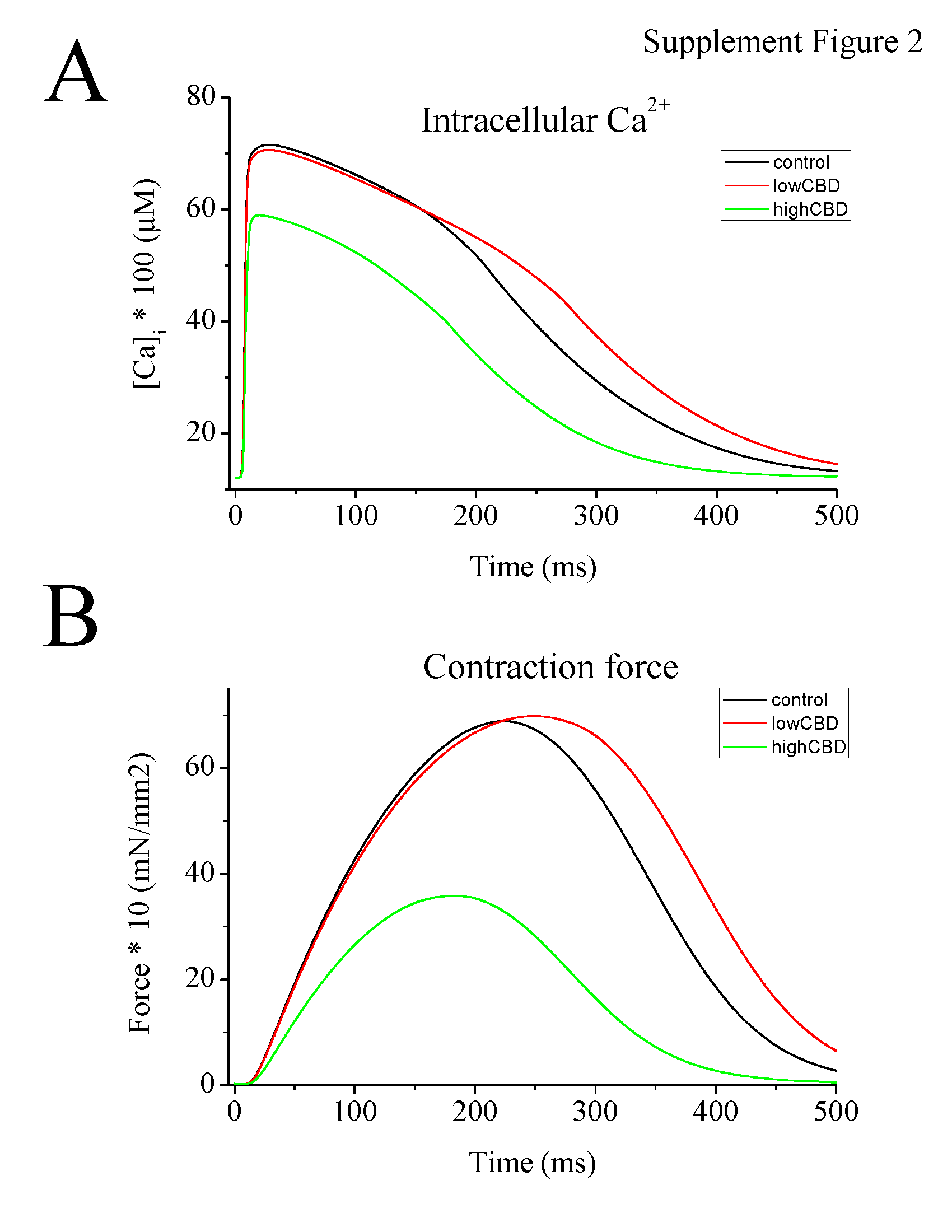

Supplement: Supplementary file 1 [file Image1.TIFF]
